# Supplementary material for: Reliability and Validity of the ONAPS Physical Activity Questionnaire in Assessing Physical Activity and Sedentary Behavior in French Adults
Source: Int J Environ Res Public Health. 2021 May 25;18(11):5643. doi: 10.3390/ijerph18115643 (PMC8197457; doi:10.3390/ijerph18115643)
Supplement: Supplementary file 1 [file ijerph-18-05643-s001.zip › ijerph-1199326-supplementary.pdf]

Questionnaire

Activité physique & Sédentarité

ONAPS – Observatoire National de l'Activité Physique et de la Sédentarité

Champ réservé expérimentateur

Code sujet : _ _ _ _ _ _ _ _ _

Ce questionnaire évalue votre niveau d'activité physique et de sédentarité au cours d'une **semaine habituelle** **(du LUNDI au DIMANCHE)**. Il est divisé en 3 parties et 21 questions (de Q1 à Q21) :

- **PARTIE A** : les activités au travail ;
- **PARTIE B** : les déplacements à but utilitaire ;
- **PARTIE C** : les activités de loisirs ou au domicile.

Quelle est la date d'aujourd'hui ?
_ _ / _ _ / _ _ _ _ (JJ/MM/AAAA)

Quel est votre âge ?
|__|__| (ans)

Précisez votre sexe :
☐ Femme ☐ Homme

Exercez-vous actuellement une activité (rémunérée ou non), ou suivez-vous des études ou une formation ?
☐ Oui ☐ Non → Ne pas remplir la PARTIE A ⚠

PARTIE A : ACTIVITÉS AU TRAVAIL

Les questions suivantes concernent tout type de travail, rémunéré ou non (y compris bénévole) ainsi que les études.

ACTIVITÉ PHYSIQUE : COMMENT RECONNAITRE L'INTENSITÉ SELON VOTRE PERCEPTION ?

❶ *Les activités physiques de **forte intensité** sont des activités nécessitant un effort physique important et causant une augmentation conséquente de la respiration ou du rythme cardiaque.*

❷ *Les activités physiques **d'intensité modérée** sont des activités qui demandent un effort physique modéré et causant une petite augmentation de la respiration ou du rythme cardiaque.*

Q1 Votre travail implique-t-il des **tâches répétitives ou régulières** :

*ⓘ si ✔ **NON** pour les deux types d'intensité, passez directement à la question Q4.*

	De forte intensité ? ❶	D'intensité modérée ? ❷
	Ex : soulever des charges lourdes…	*Ex : marcher à un rythme normal ou soutenu, soulever des charges légères…*
Q1	☐ NON ☐ OUI	☐ NON ☐ OUI

	De forte intensité ❶	D'intensité modérée ❷
**Q2** Habituellement, **combien de jours par semaine** effectuez-vous ces tâches répétitives ou régulières dans le cadre de votre travail ?	Nombre de jours : |__| *(par semaine, 7 maximum)*	Nombre de jours : |__| *(par semaine, 7 maximum)*
**Q3** Lors d'une journée durant laquelle vous effectuez ces tâches, **combien de temps en moyenne** consacrez-vous à ces tâches dans le cadre de votre travail ?	|__|__| : |__|__| heures minutes *(par jour)*	|__|__| : |__|__| heures minutes *(par jour)*

Q4 Habituellement, **combien de temps par jour en moyenne** passez-vous **assis** pour votre **travail** ?
(⚠ ne pas compter les temps de repas)

Heures : minutes : |__|__| : |__|__| *(par jour)*

SUITE À LA PAGE SUIVANTE

PARTIE B : DÉPLACEMENTS À BUT UTILITAIRE

« Aller d'un point A à un point B »

Les questions suivantes concernent la façon habituelle de vous déplacer d'un endroit à l'autre, par exemple pour aller au travail, faire des courses, aller au marché, aller chez un ami, se rendre sur un lieu de visite ou de balade…

	**À pied ?** ⓘ *trajet(s) à pied durant **au moins 10 minutes***	**À vélo ou VAE ?** ⓘ *VAE = vélo à assistance électrique*	**Autre mode de déplacement ACTIF ?** ⓘ *Ex : roller, trottinette… **requérant une propulsion par vos mouvements***
**Q5** Lors d'une semaine habituelle (week-end inclus), effectuez-vous des **déplacements :**	☐ NON ☐ OUI	☐ NON ☐ OUI	☐ NON ☐ OUI
	**À pied :**	**À vélo ou VAE :**	**Autre mode de déplacement actif :**
**Q6** Habituellement, **combien de jours par semaine en moyenne** effectuez-vous ces trajets ?	Nombre de jours : └─┘ *(par semaine, 7 max)*	Nombre de jours : └─┘ *(par semaine, 7 max)*	Nombre de jours : └─┘ *(par semaine, 7 max)*
**Q7** <u>Lors d'une journée</u> durant laquelle vous effectuez ces trajets, **combien de temps en moyenne** y consacrez-vous ?	└─┴─┘ : └─┴─┘ heures minutes *(par jour)*	└─┴─┘ : └─┴─┘ heures minutes *(par jour)*	└─┴─┘ : └─┴─┘ heures minutes *(par jour)*

ⓘ *si ✔ **NON** pour les trois types de déplacement, passez directement à la question Q8.*

Q8 Lors d'une semaine habituelle (week-end inclus), utilisez-vous des **moyens de transport motorisés*** pour certains de vos déplacements ?

** Ex : en voiture, moto, scooter, assis dans les transports en commun… (⚠ hors vélo à assistance électrique)*

☐ NON ☐ OUI

Q9 Habituellement, **combien de jours par semaine** effectuez-vous ces trajets motorisés ?

Nombre de jours : └─┘ *(par semaine, 7 max)*

Q10 <u>Lors d'une journée</u> durant laquelle vous effectuez ces trajets motorisés, **combien de temps en moyenne** durent l'ensemble de ces trajets ?

└─┴─┘ : └─┴─┘ heures minutes *(par jour de pratique)*

SUITE À LA PAGE SUIVANTE

PARTIE C : ACTIVITÉS DE LOISIRS OU AU DOMICILE

Les questions suivantes excluent les activités liées au travail et aux déplacements que vous avez déjà mentionnées.

ACTIVITÉ PHYSIQUE : COMMENT RECONNAITRE L'INTENSITÉ SELON VOTRE PERCEPTION ?

❶ *Les activités physiques de **forte intensité** sont des activités nécessitant un effort physique important et causant une augmentation conséquente de la respiration ou du rythme cardiaque.*

❷ *Les activités physiques **d'intensité modérée** sont des activités qui demandent un effort physique modéré et causant une petite augmentation de la respiration ou du rythme cardiaque.*

Q11 Lors d'une semaine habituelle (week-end inclus), effectuez-vous des **tâches ménagères au domicile, à l'intérieur ou à l'extérieur** ? *Ex : passer l'aspirateur, laver le sol, balayer, laver les vitres, tondre la pelouse…*

☐ NON ☐ OUI

Q12 Habituellement, **combien de jours par semaine** effectuez-vous ces tâches ménagères au domicile, à l'intérieur ou à l'extérieur ?

Nombre de jours : *(par semaine, 7 max)* |__|

Q13 Lors d'une journée durant laquelle vous pratiquez ces tâches ménagères au domicile, **combien de temps en moyenne** y consacrez-vous ?

|__|__| : |__|__| heures minutes *(par jour de pratique)*

Q14 Lors d'une semaine habituelle (week-end inclus), pratiquez-vous des **activités sportives** ou des **activités physiques de loisirs** :

*ⓘ si ✔ **NON** pour les deux types d'intensité, passez directement à la question Q17.*

De forte intensité ? ❶	D'intensité modérée ? ❷
Ex : courir, jouer au football, porter des charges lourdes, nager, jouer au squash, pratiquer des arts martiaux, faire de la zumba, faire du vélo à un rythme soutenu ou sur des pentes (hors VAE), etc.	*Ex : marcher à un rythme normal ou soutenu (hors trajet utilitaire), faire du vélo ou du VAE à un rythme modéré (hors trajet utilitaire), randonner, danser, jardiner, faire une séance de gym ou de renforcement musculaire, jouer au ping-pong, etc.*
☐ NON ☐ OUI	☐ NON ☐ OUI

	De forte intensité ❶	D'intensité modérée ❷
**Q15** Habituellement, **combien de jours par semaine** pratiquez-vous ces activités sportives ou ces activités de loisirs ?	Nombre de jours : |__| *(par semaine, 7 maximum)*	Nombre de jours : |__| *(par semaine, 7 maximum)*
**Q16** Lors d'une journée durant laquelle vous pratiquez ces activités sportives ou ces activités de loisirs, **combien de temps en moyenne** y consacrez-vous ?	|__|__| : |__|__| heures minutes *(par jour)*	|__|__| : |__|__| heures minutes *(par jour)*

SUITE À LA PAGE SUIVANTE

RAPPEL *Placez-vous toujours dans le cadre d'une **semaine habituelle** (du **LUNDI** au **DIMANCHE**).*

Q17 Lors d'une semaine habituelle, passez-vous du temps à des **activités assises ou allongées** (hors sommeil), **chez vous** ou lors de **vos loisirs** ?

*si ✔ **NON** pour les deux types d'activités, passez directement à la question Q20.*

	**Devant un écran ?**	**Autres que devant un écran?**
	Ex : télévision, jeux vidéo, ordinateur à but non professionnel (internet, emails…), tablette, smartphone, etc.	*Ex : lecture, dessin, discussion avec des amis, couture, etc.*
**Q17**	☐ **NON** ☐ **OUI**	☐ **NON** ☐ **OUI**

Q18 Habituellement, lors de **combien de jours par semaine** passez-vous du temps à ces activités assises ou allongées, chez vous ou lors de vos loisirs ?

Q19 Lors d'une journée durant laquelle vous passez du temps à ces activités assises ou allongées, **combien de temps en moyenne** y passez-vous, chez vous ou lors de vos loisirs ?

	**Devant un écran ?**	**Autres que devant un écran ?**
**Q18**	Nombre de jours : └─┘ *(par semaine, 7 maximum)*	Nombre de jours : └─┘ *(par semaine, 7 maximum)*
**Q19**	└─┴─┘ heures : └─┴─┘ minutes *(par jour)*	└─┴─┘ heures : └─┴─┘ minutes *(par jour)*

Q20 Veuillez **préciser la nature du ou des sport(s)** que vous pratiquez lors de vos loisirs, en fonction de leur **fréquence** de pratique :

⊕⊕⊕ Au moins **3 fois** par **semaine** ?	⊕⊕ **1 à 2 fois** par **semaine** ?	⊕ **Plus d'1 fois** par **mois**, mais **moins d'1 fois** par **semaine** ?
Veuillez inscrire le ou les sport(s) pratiqué(s)	*Veuillez inscrire le ou les sport(s) pratiqué(s)*	*Veuillez inscrire le ou les sport(s) pratiqué(s)*
………………………………	………………………………	………………………………
………………………………	………………………………	………………………………
………………………………	………………………………	………………………………
☐ Aucun	☐ Aucun	☐ Aucun

Q21 Quelle est votre **catégorie socioprofessionnelle** ?

Veuillez ne cocher qu'une seule case ; si actuellement au chômage, veuillez cocher votre catégorie antérieure.

- ☐ Agriculteurs exploitants
- ☐ Artisans, commerçants et chefs d'entreprise
- ☐ Employés (employé de bureau ou de commerce, garde d'enfants, agent de service…)
- ☐ Cadres et professions intellectuelles supérieures (ingénieur, médecin…)
- ☐ Ouvriers (y compris agricoles)
- ☐ Professions intermédiaires (professeur des écoles, infirmier, assistant social, technicien, contremaître…)
- ☐ Retraités
- ☐ Étudiants
- ☐ Autres inactifs (par exemple parent au foyer)

FIN
